# Serum organic acid metabolites can be used as potential biomarkers to identify prostatitis, benign prostatic hyperplasia, and prostate cancer

**DOI:** 10.3389/fimmu.2022.998447

**Published:** 2023-01-04

**Authors:** Jinhua He, Zeping Han, Wenfeng Luo, Jian Shen, Fangmei Xie, Liyin Liao, Ge Zou, Xin Luo, Zhonghui Guo, Yuguang Li, Jianhao Li, Hanwei Chen

**Affiliations:** ^1^ Central Laboratory, Central Hospital of Panyu District, Guangzhou, China; ^2^ Urinary Surgery Department, Central Hospital of Panyu District, Guangzhou, China; ^3^ He Xian Memorial Hospital, Southern Medical University, Guangzhou, China; ^4^ Institute of Cardiovascular Medicine, Central Hospital of Panyu District, Guangzhou, China; ^5^ Medical Imaging Institute, Central Hospital of Panyu District, Guangzhou, China

**Keywords:** organic acid metabolites, LC-MS, prostatitis, benign prostatic hyperplasia, prostate cancer

## Abstract

**Background:**

Noninvasive methods for the early identify diagnosis of prostatitis, benign prostatic hyperplasia (BPH), and prostate cancer (PCa) are current clinical challenges.

**Methods:**

The serum metabolites of 20 healthy individuals and patients with prostatitis, BPH, or PCa were identified using untargeted liquid chromatography-mass spectrometry (LC-MS). In addition, targeted LC-MS was used to verify the organic acid metabolites in the serum of a validation cohort.

**Results:**

Organic acid metabolites had good sensitivity and specificity in differentiating prostatitis, BPH, and PCa. Three diagnostic models identified patients with PROSTATITIS: phenyllactic acid (area under the curve [AUC]=0.773), pyroglutamic acid (AUC=0.725), and pantothenic acid (AUC=0.721). Three diagnostic models identified BPH: citric acid (AUC=0.859), malic acid (AUC=0.820), and D-glucuronic acid (AUC=0.810). Four diagnostic models identified PCa: 3-hydroxy-3-methylglutaric acid (AUC=0.804), citric acid (AUC=0.918), malic acid (AUC=0.862), and phenyllactic acid (AUC=0.713). Two diagnostic models distinguished BPH from PCa: phenyllactic acid (AUC=0.769) and pyroglutamic acid (AUC=0.761). Three diagnostic models distinguished benign BPH from PROSTATITIS: citric acid (AUC=0.842), ethylmalonic acid (AUC=0.814), and hippuric acid (AUC=0.733). Six diagnostic models distinguished BPH from prostatitis: citric acid (AUC=0.926), pyroglutamic acid (AUC=0.864), phenyllactic acid (AUC=0.850), ethylmalonic acid (AUC=0.843), 3-hydroxy-3-methylglutaric acid (AUC=0.817), and hippuric acid (AUC=0.791). Three diagnostic models distinguished PCa patients with PROSTATITISA < 4.0 ng/mL from those with PSA > 4.0 ng/mL: 5-hydromethyl-2-furoic acid (AUC=0.749), ethylmalonic acid (AUC=0.750), and pyroglutamic acid (AUC=0.929). Conclusions: These results suggest that serum organic acid metabolites can be used as biomarkers to differentiate prostatitis, BPH, and PCa.

## Introduction

Prostate diseases are common disease in adult men and usually refers to benign prostatic hyperplasia (BPH), prostatitis, and prostate cancer(PCa) (1). At present, the auxiliary diagnostic examinations for prostatitis, prostatic hyperplasia, and prostate cancer mainly include digital rectal examination, ultrasound, X-ray examination, pathological tissue biopsy, mechanical examination of urinary activity in the lower urinary tract, routine prostatic fluid tests, computed tomography examination, and magnetic resonance imaging examination. Serum markers include prostate-specific antigen (PSA). These tests and markers have certain deficiencies (2), and there are currently no efficient and convenient method or serum biomarkers with strong specificity and sensitivity. However, the development of metabolomics in recent years has started to bridge this gap and is now playing an important role in the auxiliary diagnosis of diseases.

Metabolomics is a discipline that complements genomics, proteomics and transcriptomics integrates systems mainly through high-throughput detection and information modeling and data processing (3). Metabolomics analytical techniques can identify metabolites with molecular weights lower than 1000 D, such as vitamins, lipids and sugars. The pathophysiological state of the corresponding organism in a certain period can be determined by changes in metabolic levels, and the small-molecule metabolites in this state can be used as biomarkers, providing effective help for the screening and early diagnosis of diseases (4, 5). Metabolomics analytical techniques mainly comprise nuclear magnetic resonance combined with mass spectrometry (MS) and liquid chromatography-mass spectrometry (LC-MS) technologies (6). LC-MS has been widely used in the analysis of biological fluid samples owing to its high resolution, high sensitivity, and high selectivity (7).

In the present study, we used untargeted and targeted LC-MS techniques to study metabolites in the serum of normal individuals and patients with prostatitis, BPH, or PCa in the hope of identifying a new biomarker and providing important clues for the early detection and rapid diagnosis of the disease.

## Methods and materials

### Inclusion criteria

Prostatitis, prostatic hyperplasia (BPH),and prostate cancer(PCa) were diagnosed according to European Association of Urology guidelines (8). Participants were classified as follows: (i) healthy individuals (control group), with normal findings on digital rectal examination and prostate B-mode ultrasound and excluding those with diseases of the urinary system, all kinds of malignant tumors, and all kinds of chronic diseases; (ii) prostatitis patients (prostatitis group), with typical clinical symptoms of urination urgency, frequency, and pain, with abnormal results on a routine examination of prostatic fluid, and no abnormalities in liver and kidney function and no drug treatment or surgical resection; (iii) BPH group, with digital rectal examination and B-mode ultrasound of the prostate showing increased prostate volume and the presence of poor urination, frequent urination, increased nocturia, and progressive dysuria; some patients may have a history of urinary retention, but none have a history of other malignant tumors; all patients underwent urethral resection of the prostate and the postoperative pathological diagnosis confirmed BPH; and (iv) prostate cancer group, with serum PSA, digital rectal examination, prostate ultrasound, multiparametric magnetic resonance scanning, and other examinations confirming the diagnosis of prostate biopsy pathology; other systemic diseases were excluded and there was no history of other malignant tumors; finally, no patients were treated with radiotherapy, chemotherapy, surgical castration, drug castration, or surgical resection. All participants provided signed informed consent. The study was approved by the Ethics Committee of Panyu District Central Hospital in Guangzhou.

### Specimen collection

Preoperatively, 3 mL of fasting venous blood was taken from all participants (digital rectal examination, massage, puncture, and other exploratory procedures were forbidden 1 week before blood collection), and the serum was collected and stored at −80°C for analysis. Patient information and relevant laboratory test indicators were collected retrospectively from patient records.

### Metabolomics analysis

Based on ultra-high-performance liquid chromatography mass spectrometry (UHPLC-MS) platform analysis, serum extracts were obtained using methanol, and organic acids were analyzed by UHPLC-triple quadruple mass spectrometry. Substances identified by non-targeted liquid chromatography mass spectrometry (LC-MS) metabolomics include, organic acids, nucleotides, fatty acids, lipids, amino acids and other substances. The original MS raw file was converted to mzXML file format by the msConvert tool in ProteoWizard software package (V3.0.8789). The RXCMS software package was used for peak detection, peak filtering, and peak alignment, and a quantitative list of substances was obtained. The substances were identified using the HMDB (Human Metabolome Database), Metlin, MassBank, Lipid Maps, mzCloud, and KEGG(Kyoto Encyclopedia of Genes and Genomes) and a self-built database. The lose signal correction method based on quality control samples can conduct data correction and eliminate systematic errors. We filtered out material with a relative standard deviation>30% in quality control samples in data quality control.

## Results

### Characteristics of the study population

The characteristics of the participants in the discovery cohort are summarized in **Table 1**. Total, free, and complexed PSA contents were all higher in the prostatitis group, BPH group, and prostate cancer group than in the normal control group. Although the mean age of the patients in the control group was lower than that in the other groups, the serum metabolic profile of the samples did not differ due to age, as shown by unsupervised principle component analysis in **Supplemental Material, Figures 1**-**3**. The characteristics of the participants in the validation cohort are summarized in **Table 2** and were generally very similar to those of the discovery cohort. As shown in **Supplemental Material, Figures 4**-**6**, the samples of the patient and healthy control groups in the verification cohort could be separated and the same group was clustered together, indicating good repeatability within the group.

**Table 1:** Comparison of PSA levels found in the discovery cohort.

	Control	prostatitis	BPH	PC
n	20	20	20	20
Age (years)	32.45±5.8	60.65±7.5*	53.25±8*	67.05±7.8*
TPSA	0.90±0.1	4.03±0.6*	3.31±2.5*	66.83±0.7*
FPSA	0.26±0.1	0.45±0.3*	0.58±1.1*	8.15±0.4*
CPSA	0.65±0.2	3.59±0.4*	2.73±2.2	58.68±0.6*
CPSA/TPSA	0.72±0.1	0.75±0.8	0.77±0.9	0.76±0.7
FPSA/TPSA	0.28±0.1	0.25±0.6	0.22±0.4	0.24±0.6

BPH, benign prostatic hyperplasia; CPSA, complexed prostate-specific antigen; FPCS, free prostate-specific antigen; PC, prostate cancer; PSA, prostate-specific antigen; TPSA, total prostate-specific antigen.

*p<0.05, compared with the control group.

**Table 2:** Comparison of PSA levels in the validation cohort.

	Control	Prostatitis	BPH	PC
n	38	37	52	52
Age (years)	38.82±9.29	52.05±12.35*	66.08±11.02*	77.81±8.49*
TPSA	0.74±0.37	1.7±2.49	11.32±39.31*	181.12±1212.22*
FPSA	0.25±0.11	0.34±0.14	1.44±2.56	215.31±1267.55*
CPSA	0.48±0.31	0.98±0.95	9.06±36.89*	32.9±153.69*
CPSA/TPSA	0.63±0.11	0.67±0.15	0.96±1.09	0.73±0.24
FPSA/TPSA	0.37±0.11	1.97±1.06	2.31±3.28	19.32±29.76*

BPH, benign prostatic hyperplasia; CPSA, complexed prostate-specific antigen; FPCS, free prostate-specific antigen; PC, prostate cancer; PSA, prostate-specific antigen; TPSA, total prostate-specific antigen.

*p<0.05, compared with the control group.

### Analysis of serum metabolites found in the discovery cohort

Non-targeted LC-MS technology was used to identify prostatitis, and 20 metabolites with an AUC value of 1 were obtained, including organic heterocyclic compounds, organic oxygen compounds, lipids and lipid-like molecules, organic acids and derivatives, and phenylpropanoids and polypeptides (**Supplemental Material, Table 1**). There were nine metabolites with an AUC value of 1, which were mainly organic heterocyclic compounds, lipids and lipid-like molecules, and organic nitrogen compounds (**Supplemental Material, Table 2**). A total of 33 metabolites with an AUC value of 1 were obtained for identifying prostate cancer, including phenylpropanoids and polypeptides, lipids and functional-like molecules, organic acids and derivatives, organic oxygen compounds, organic heterocyclic compounds, and benzenoids (**Supplemental Material, Table 3**). The results showed that metabolites of organic acids in the peripheral blood of patients with prostatitis, BPH, and prostate cancer had good sensitivity and specificity for identifying the respective condition.

### Validation analysis of serum organic acid metabolites in the validation cohort

**Figure 1:**
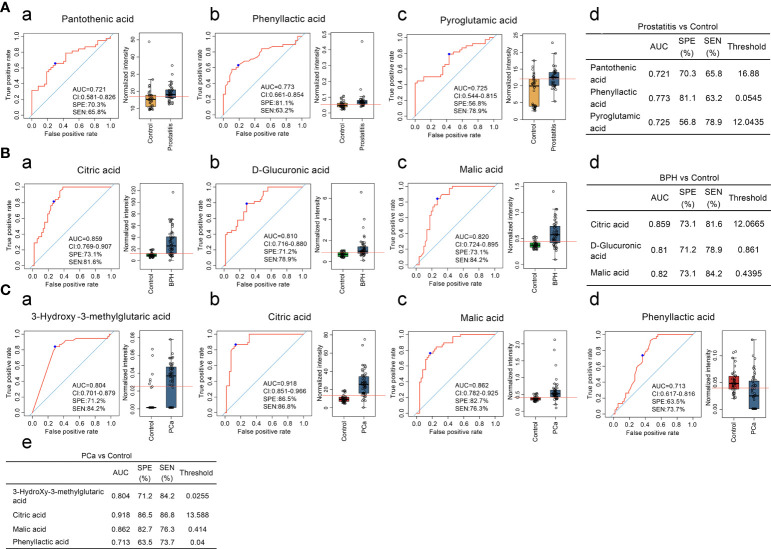
**(A)** Comparison of the contents of organic acid metabolites in the serum of prostatitis and normal controls and ROC curve analysis. (a, b) The contents of pantothenic acid, phenyllactic acid, and pyroglutamic acid in the serum of prostatitis patients and normal controls and ROC curve analysis of lactic acid, used to identify prostatitis. (d) Diagnostic model for identifying prostatitis with serum organic acids. **(B)** Comparison of the contents of organic acid metabolites in the serum of patients with BPH and normal controls and ROC curve analysis. (a–c) Content of citric acid, D-glucuronic acid, and malic acid in the serum of patients with BPH and ROC curve analysis for differentiating BPH. (d) Diagnostic model of serum organic acid metabolites for differentiating BPH. **(C)** Comparison of the contents of organic acid metabolites in the serum of prostate cancer patients and normal controls and ROC curve analysis. (a–d) The contents of 3-hydroxy-3-methylglutaric acid, citric acid, malic acid, and phenyllactic acid in the serum of prostate cancer patients and normal controls and ROC curve analysis of lactic acid, used to identify prostate cancer. (e) Diagnostic model of serum organic acid metabolites for differentiating prostate cancer.

Three diagnostic models were obtained for identifying prostatitis, with sensitivities and specificities of 63.2% and 81.1% for phenyllactic acid, 78.9% and 56.8% for pyroglutamic acid, and 65.8% and 70.3% for pantothenic acid, respectively. The serum levels of phenyllactic acid, pyroglutamic acid, and pantothenic acid were significantly higher in the prostatitis group than in the normal control group (**Figure 1A**).

Three diagnostic models were obtained for the differential diagnosis of BPH, with sensitivities and specificities of 81.6% and 73.1% for citric acid, 84.2% and 73.1% for malic acid, and 78.9% and 71.2% for D-glucuronic acid, respectively. The serum contents of citric acid, malic acid, and D-glucuronic acid were significantly higher in the BPH group than in the normal control group (**Figure 1B**).

Four diagnostic models were used to identify prostate cancer, with sensitivities and specificities of 86.8% and 86.5% for citric acid, 76.3% and 82.7% for malic acid, 84.2% and 71.2% for 3-hydroxy-3-methylalutaric acid, 73.7% and 63.5% for lactic acid, respectively. As the degree of malignancy increased, the sensitivity and specificity of citric acid and malic acid also increased, suggesting that these metabolites can be used as good biomarkers for monitoring malignant prostatic hyperplasia (**Figure 1C**).

**Figure 2:**
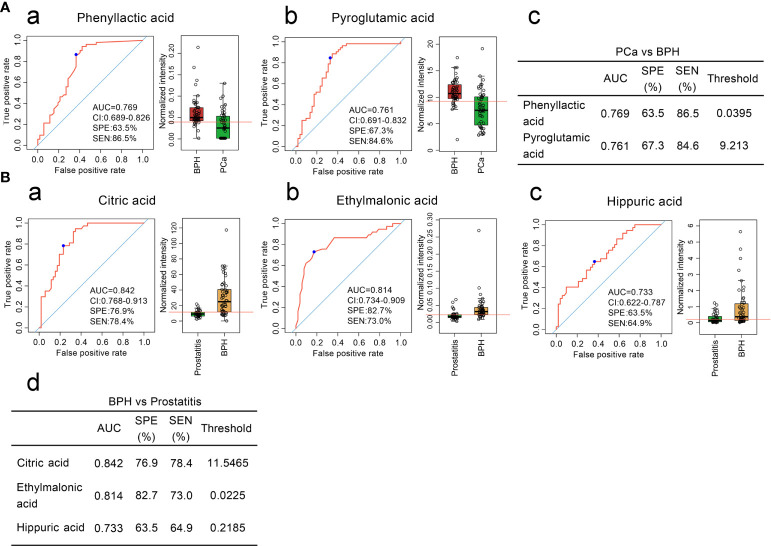
**(A)** Comparison of the contents of organic acid metabolites in the serum of patients with BPH and patients with prostate cancer and ROC curve analysis. (a, b) Comparison of the contents of phenyllactic acid and pyroglutamic acid in the serum of patients with BPH and ROC curve analysis. (c) Diagnostic model of the serum organic acid metabolites used to distinguish prostate cancer from BPH. **(B)**. Comparison of the contents of organic acid metabolites in the serum of patients with BPH and patients with prostatitis and ROC curve analysis. (a–c) Comparison of the contents of citric acid, ethylmalonic acid, and hippuric acid in the serum of patients with BPH and patients with prostatitis and ROC curve analysis. (d) Diagnostic model of serum organic acid metabolites to distinguish BPH from prostatitis.

To further distinguish benign and malignant prostatic hyperplasia, two diagnostic models were obtained through ROC curve analysis. The sensitivity and specificity of phenyllactic acid were 86.5% and 63.5% while those of pyroglutamic acid were 84.6% and 67.3%, respectively (**Figure 2A**).

Three diagnostic models were obtained to distinguish BPH and prostatitis. The sensitivities and specificities were 78.4% and 76.9% for citric acid, 73.0% and 82.7% for ethylmalonic acid, and 64.9% and 63.5% for hippuric acid, respectively (**Figure 2B**).

**Figure 3:**
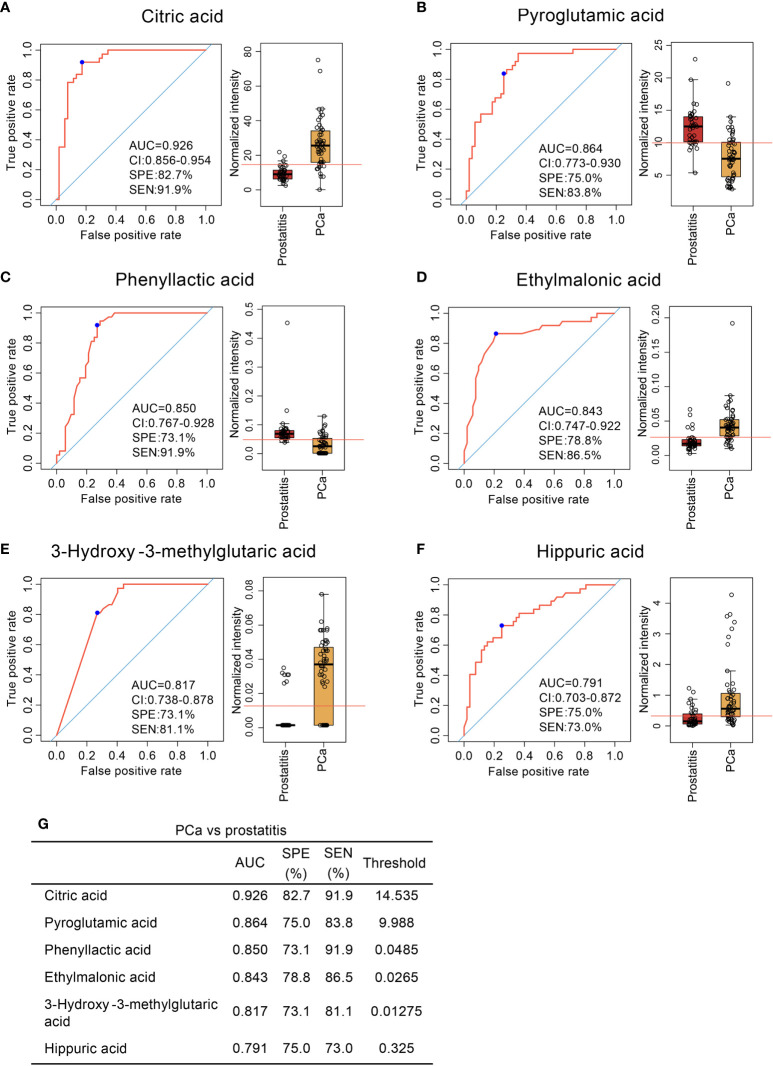
Comparison of organic acid metabolites in the serum of patients with prostate cancer and patients with prostatitis and ROC curve analysis. **(A–F)** Comparison of citric acid, pyroglutamic acid, phenyllactic acid, ethylmalonic acid, 3-hydroxy-3-methylglutaric acid, and hippuric acid content in the serum of patients with prostate cancer and patients with prostatitis and ROC curve analysis. **(G)** Diagnostic models of serum organic acid metabolites used to distinguish prostate cancer from prostatitis.

Six diagnostic models were obtained for distinguishing prostate cancer from prostatitis. The sensitivities and specificities were 91.9% and 82.7% for citric acid, 83.8% and 75.0% for pyroglutamic acid, 91.9% and 73.1% for phenyllactic acid. 86.5% and 78.8% for ethylmalonic acid, 81.1% and 73.1% for 3-hydroxy-3-methylglutaric acid, and 73.0% and 75.0% for hippuric acid, respectively (**Figure 3**). Thus, serum organic acid metabolites can be used as potential biomarkers to differentiate prostatitis, BPH, and prostate cancer.

### Analysis of organic acid metabolites in the serum of PCa patients

**Figure 4:**
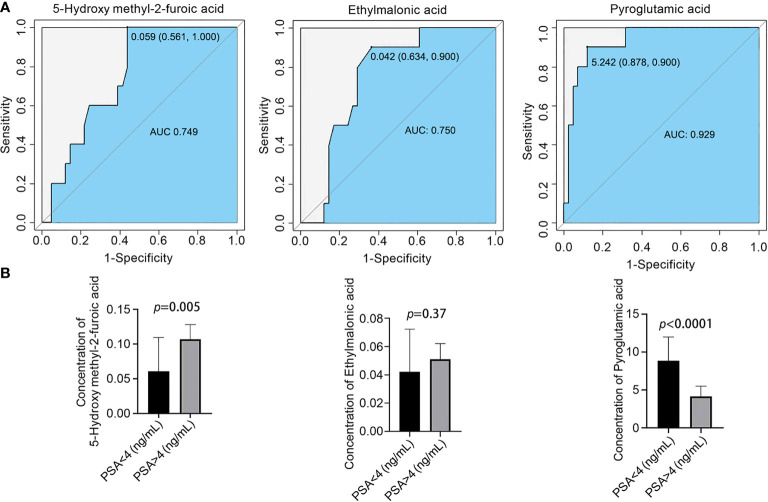
Comparison of the contents of organic acid metabolites in the serum of different prostate cancer groups and ROC curve analysis. **(A)** ROC curve analysis of 5-hydroxymethyl-2-furoic acid, ethylmalonic acid, and pyroglutamic acid for distinguishing prostate cancer patients with PSA < 4.0 ng/mL and PSA > 4.0 ng/mL. **(B)** Comparison of 5-hydroxymethyl-2-furoic acid, ethylmalonic acid, and pyroglutamic acid in the serum of prostate cancer patients with PSA < 4.0 ng/mL and PSA > 4.0 ng/mL.

PSA is a specific tumor marker for prostate cancer that is mainly used for the auxiliary diagnosis of PCa. When the serum total PSA is higher than 4.0 ng/mL, prostate cancer is highly suspected. However, the serum PSA content of some prostate cancer patients is less than 4.0 ng/mL, and the definitive diagnosis of prostate cancer should be combined with clinical imaging and pathological examinations (9, 10). To further distinguish prostate cancer patients with PSA < 4.0 ng/mL from those with PSA < 4.0 ng/mL, ROC curve analysis was used to obtain three diagnostic models: the sensitivities and specificities were 56.1% and 100% for 5-hydroxymethyl-2-furoic acid, 63.4.0% and 100% for ethylmalonic acid, and 87.8% and 90% for pyroglutamic acid, respectively (**Figure 4A**). The serum content of pyroglutamic was higher in patients with PSA < 4.0 ng/mL than in patients with PSA > 4.0 ng/mL. The serum content of 5-hydroxymethyl-2-furoic acid was significantly higher in patients with PSA > 4.0 ng/mL than in patients with PSA < 4.0 ng/mL (**Figure 4B**). These results showed that 5-hydroxymethyl-2-furoic acid, ethylmalonic acid, and pyroglutamic acid could be used as biomarkers to differentiate prostate cancer with PSA < 4.0 ng/mL from that with PSA > 4.0 ng/mL.

## Discussion

Increasing evidence indicates that the onset and progression of disease may influence the release of specific metabolites from the humeral microenvironment (11, 12). Serum sarcosine has been identified as a biomarker of invasive prostate cancer. It increases significantly during prostate cancer progression to metastasis and can be detected in urine, and increased sarcosine levels have been found in invasive prostate cancer cell lines relative to benign prostate epithelial cells (13). N-acetyl-3-methylhistidine has been implicated in the progression of prostate cancer (14). Accurate identification of noninvasive biomarkers remains a challenge. To date, few plasma/serum metabolomics have been used to identify prostate diseases, including prostatitis, BPH, and prostate cancer.

In our study, 26 types of serum organic metabolites were identified based on targeted LC-MS technology. Three diagnostic models were found to identify patients with prostatitis: phenyllactic acid (AUC=0.773), pyroglutamic acid (AUC=0.725), and pantothenic acid (AUC=0.721). Three diagnostic models identified BPH: citric acid (AUC=0.859), malic acid (AUC=0.820), and D-glucuronic acid (AUC=0.810). Four diagnostic models identified prostate cancer: 3-hydroxy-3-methylglutaric acid (AUC=0.804), citric acid (AUC=0.918), malic acid (AUC=0.862), and phenyllactic acid (AUC=0.713). Two diagnostic models distinguished BPH from prostate cancer: phenyllactic acid (AUC=0.769) and pyroglutamic acid (AUC=0.761). Three diagnostic models distinguished BPH from prostatitis: citric acid (AUC=0.842), ethylmalonic acid (AUC=0.814), and hippuric acid (AUC=0.733). Six diagnostic models were obtained for distinguishing prostate cancer from prostatitis: citric acid (AUC=0.926), pyroglutamic acid (AUC=0.864), phenyllactic acid (AUC=0.850), ethylmalonic acid (AUC=0.843), 3-hydroxy-3-methylglutaric acid (AUC=0.817), and hippuric acid (AUC=0.791). Three diagnostic models distinguished prostate cancer patients with PSA < 4.0 ng/mL from those with PSA < 4.0 ng/mL: 5-hydroxymethyl-2-furoic acid (AUC=0.749), ethylmalonic acid (AUC=0.750), and pyroglutamic acid (AUC=0.929). Therefore, serum organic acid metabolites can be used as biomarkers to differentiate prostatitis, BPH, and prostate cancer.

The serum marker PSA is a glycoprotein produced by intracytoplasmic vesicles in prostate epithelial cells. When prostate disease occurs, the tissue barrier between prostate vesicles and the ductal lumen and the blood circulation system is damaged to varying degrees, resulting in leakage of PSA protein into the blood and an elevated PSA concentration (15). PSA levels can be increased by prostate diseases such as prostatitis, prostatic hyperplasia, and prostate ischemia and prostate stimulation such as anal finger examination, prostate massage, cystoscopy, and acute urinary retention. When prostate cancer occurs, the original tissue barrier is severely damaged due to the abnormal infiltration and growth of cancer tissue, resulting in massive leakage of PSA into the blood. PSA is a sensitive marker for the diagnosis of prostate cancer but does not have specificity (10). To further distinguish prostate cancer patients with PSA > 4.0 ng/mL and PSA < 4.0 ng/mL, our results confirmed that the serum content of pyroglutamic was higher in patients with PSA < 4.0 ng/mL than in patients with PSA > 4.0 ng/mL. The serum content of 5-hydroxymethyl-2-furoic acid was significantly higher in the PSA > 4.0 ng/mL group than in the PSA < 4.0 ng/mL group. Thus, 5-hydroxymethyl-2-furoic acid, ethylmalonic acid, and pyroglutamic acid can be used to distinguish prostate cancer with PSA < 4.0 ng/mL and PSA > 4.0 ng/mL with high sensitivity and specificity.

The commonly used examination methods for clinical prostatitis include digital rectal examination, routine examination of prostatic fluid, and cumbersome diagnosis methods (16). Anal examination, B-mode ultrasound, diffusion-weighted magnetic resonance imaging, and urodynamics are somewhat helpful for the diagnosis of prostatic hyperplasia, but they lack specificity and sensitivity (17). Clinical methods used for prostate cancer diagnosis, such as transrectal ultrasound prostate examination and transrectal prostate puncture biopsy, are often invasive and cause great harm to the human body. PSA is not completely applicable to the diagnosis of prostate diseases (18). The detection of serum organic acid metabolites by LC/MC technology has potential clinical application value in the identification of prostatitis, prostatic hyperplasia, and prostate cancer, which can reduce patients’ puncture pain and compensate for the deficiency of PSA in the diagnosis of prostate diseases (**Supplemental Material, Figure 7**).

In conclusion, serum metabolomics analysis is a promising noninvasive method for the diagnosis of prostatitis, BPH, and PCa and can distinguish patients with prostatitis from those with BPH and PCa. Larger validation studies in patients with different conditions and ethnicities will be needed to further determine the clinical diagnostic value of these biomarkers.

## Data availability statement

The original contributions presented in the study are publicly available. The names of the repository/repositories and accession number(s) [metabolic bank repository under the accession numbers MTBLS6038; MTBLS6039] can be found in the article/supplementary material. Further inquiries can be directed to the corresponding author.

## Ethics statement

The studies involving human participants were reviewed and approved by the ethics committee of Panyu District Central Hospital of Guangzhou. Written informed consent was obtained from all patients. The patients/participants provided their written informed consent to participate in this study.

## Author contributions

JHH, ZPH, and JS wrote the main manuscript text. FMX and LYL prepared figures. GZ and XL collected the samples. ZHG, YGL, JHL, and HWC offer the fundings. All authors contributed to the article and approved the submitted version.

## Funding

This work was supported by Medical Science and Technology Research Project of Guangdong Province (No. A2022524; A2020304), Science and Technology Program of Guangzhou (No. 202201010840; 202201010810; 202102080532; 202002030032; 202002020023), Health Commission Program of Guangzhou (20212A010025; 20201A010085), Science and Technology Project of Panyu, Guangzhou (2022-Z04-009; 2022-Z04-090; 2022-Z04-072; 2021-Z04-053; 2020-Z04-026; 2019-Z04-02), Scientific Research project of Guangzhou Panyu Central Hospital (No. 2022Y002; 2021Y004; 2021Y002).

## Conflict of interest

The authors declare that the research was conducted in the absence of any commercial or financial relationships that could be construed as a potential conflict of interest.

## Publisher’s note

All claims expressed in this article are solely those of the authors and do not necessarily represent those of their affiliated organizations, or those of the publisher, the editors and the reviewers. Any product that may be evaluated in this article, or claim that may be made by its manufacturer, is not guaranteed or endorsed by the publisher.

## References

[1] Zhang L Wang Y Qin Z. Correlation between prostatitis, benign prostatic hyperplasia and prostate cancer: A systematic review and meta-analysis. J Cancer (2021) 11(1):177–89. 10.7150/jca.37235 PMC6930406 31892984

[2] Gonzalez E Liempd SV Conde-Vancells J. Serum UPLC-MS/MS metabolic profiling in an experimental model for acute-liver injury reveals potential biomarkers for hepatotoxicity. Metabolomics (2012) 8(6):997–1011. doi: 10.1007/s11306-011-0329-9 PMC3490499 23139648

[3] O’Connell TM. Recent advances in metabolomics in oncology. Bioanalysis (2012) 4(4):431–51. doi: 10.4155/bio.11.326 22394143

[4] Beale DJ Pinu FR Kouremenos KA. Review of recent developments in GC–MS approaches to metabolomics-based research. Metabolomics (2018) 14(11). doi: 10.1007/s11306-018-1449-2 30830421

[5] Kdadra M Höckner S Leung H Kremer W. Metabolomics biomarkers of prostate cancer: A systematic review. Diagnost (Basel Switzerland) (2019) 9(1):21. doi: 10.3390/diagnostics9010021 PMC6468767 30791464

[6] Xu Y-J Liu Y. Metabolomics to Study the Therapeutic Value of Natural Compounds to Treat Obesity. Eds. Ifuentes A Comprehensive Foodomics, Elsevier (2021) 579–592. doi: 10.1016/B978-0-08-100596-5.22885-0

[7] Gakis G Bruins HM Cathomas R Compérat Witjes EM AJ. European association of urology guidelines on primary urethral carcinoma-2020 update. European Urology Oncology (2020) 3(4): 424–432. doi: 10.1016/j.euo.2020.06.003 32605889

[8] Zheng Z Zhou Z Yan W. Tumor characteristics, treatments, and survival outcomes in prostate cancer patients with a PSA level. BMC Cancer (2020) 20(1). doi: 10.1186/s12885-020-06827-z PMC7178745 32321456

[9] Liu J Dong B Qu W. Using clinical parameters to predict prostate cancer and reduce the unnecessary biopsy among patients with PSA in the gray zone. Sci Rep (2020) 10(1). doi: 10.1038/s41598-020-62015-w PMC7083895 32198373

[10] Gonzalez-Granda A Seethaler B Haap M. Effect of an intensified individual nutrition therapy on serum metabolites in critically ill patients – a targeted metabolomics analysis of the ONCA study. Clin Nutr ESPEN (2021) 43(1). doi: 10.1016/j.clnesp.2021.04.002 34024526

[11] Bai Q He B Cai Y. Transcriptomic and metabolomic analyses reveal several critical metabolic pathways and candidate genes involved in resin biosynthesis in pinus massoniana. Mol Genet Genomics (2020) 295(1). doi: 10.1007/s00438-019-01624-1 31735985

[12] Sreekumar A Poisson LM Rajendiran TM Khan AP Cao Q Yu J. Metabolomic profiles delineate potential role for sarcosine in prostate cancer progression. Nature (2009) 457(7231):910–4. doi: 10.1038/nature07762 PMC2724746 19212411

[13] Huang J Mondul AM Weinstein SJ Karoly ED Sampson JN Albanes D. Prospective serum metabolomic profile of prostate cancer by size and extent of primary tumor. Oncotarget (2017) 8(28):45190–9. doi: 10.18632/oncotarget.16775 PMC5542177 28423352

[14] Rowe E Laniado ME Walker MM. Prostate cancer detection in men with a ‘normal’ total prostate-specific antigen (PSA) level using percentage free PSA: A prospective screening study. Bju Int (2015) 95. 10.1111/j.1464-410X.2005.05514.x 15892810

[15] Khan FU Ihsan AU Khan HU. Comprehensive overview of prostatitis. Biomed Pharmacother = Biomed Pharmacother (2017) 94:1064. doi: 10.1016/j.biopha.2017.08.016 28813783

[16] Bach T Heitz M Bruns T. Benign prostatic hyperplasia: New and treatment-relevant aspects from the DGU guidelines. Der Urol (2020) 59(5):544–9. doi: 10.1007/s00120-020-01184-y 32274543

[17] Shenhar C Degani H Ber Y Baniel J Tamir S Benjaminov O. Diffusion is directional: innovative diffusion tensor imaging to improve prostate cancer detection. Diagnostics (Basel, Switzerland) (2021) 11(3): 563. doi: 10.3390/diagnostics11030563 33804783 PMC8003841

[18] Lokant MT Naz RK. Presence of PSA auto-antibodies in men with prostate abnormalities (prostate cancer/benign prostatic hyperplasia/prostatitis). Andrologia (2015) 47(3). doi: 10.1111/and.12265 24620795

