# Supplementary material for: Serum organic acid metabolites can be used as potential biomarkers to identify prostatitis, benign prostatic hyperplasia, and prostate cancer
Source: Front Immunol. 2023 Jan 4;13:998447. doi: 10.3389/fimmu.2022.998447 (PMC9846500; doi:10.3389/fimmu.2022.998447)
Supplement: Supplementary file 1 [file DataSheet_1.docx]

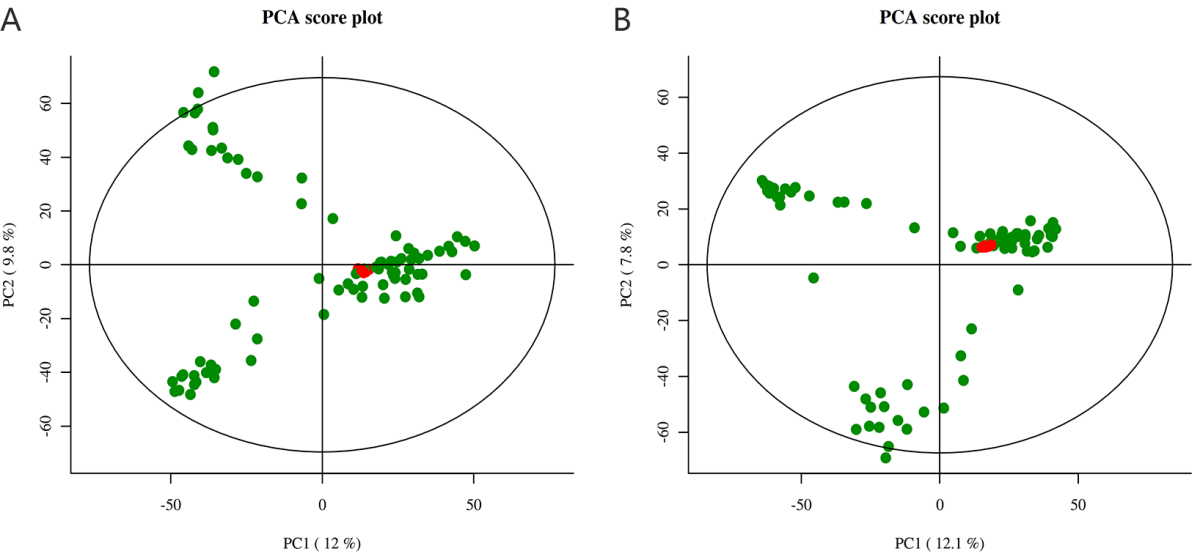


supplementary material ,Figure 1. Principal Component Analysis scoring chart of sample quality control in positive and negative ion mode

A: Quality control samples in positive-ion mode Principal Component Analysis score; B: Quality control sample in negative ion mode Principal Component Analysis score
