# Supplementary material for: Serum organic acid metabolites can be used as potential biomarkers to identify prostatitis, benign prostatic hyperplasia, and prostate cancer
Source: Front Immunol. 2023 Jan 4;13:998447. doi: 10.3389/fimmu.2022.998447 (PMC9846500; doi:10.3389/fimmu.2022.998447)
Supplement: Supplementary file 2 [file DataSheet_2.docx]

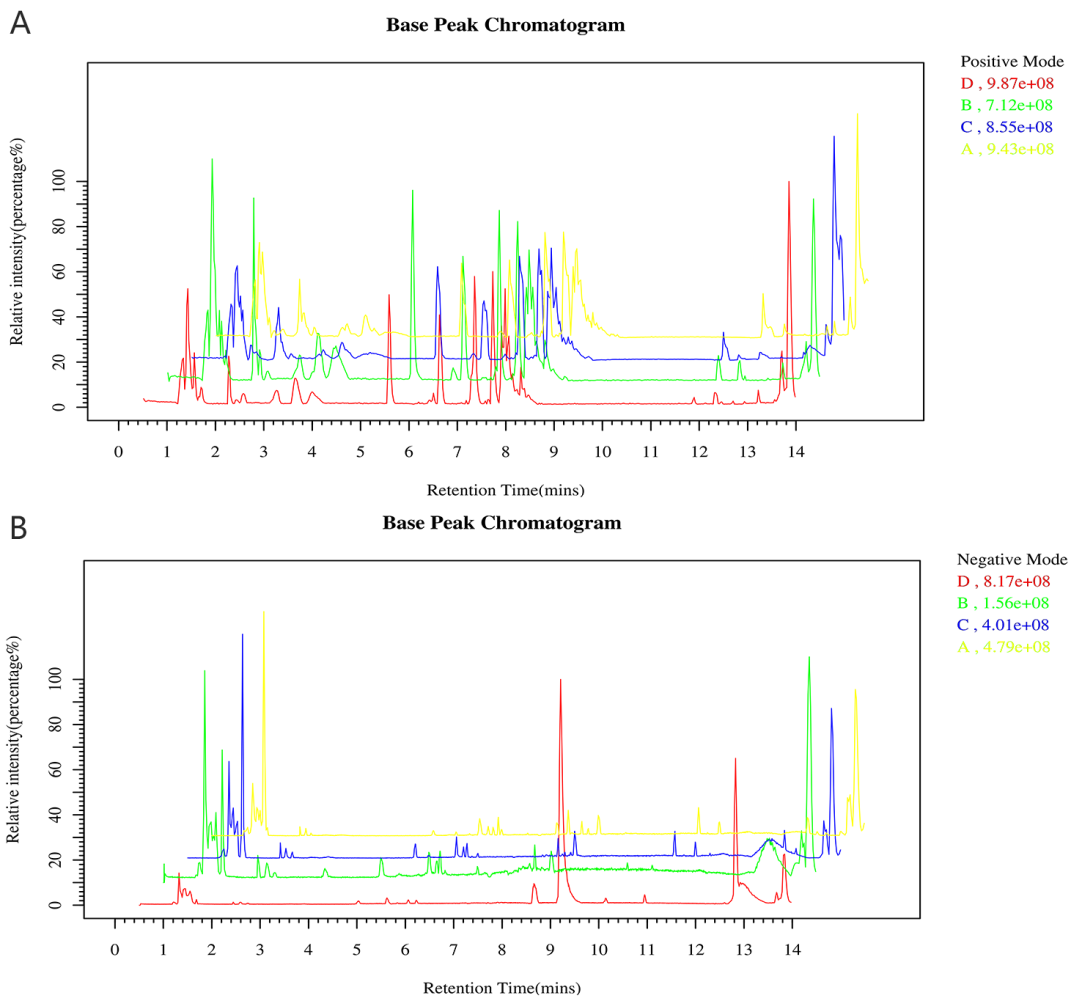


Supplemental materials, Figure 2. Basic peak chromatogram of typical sample

A: Peak Chromatogram of typical samples in positive ion mode

B: Peak Chromatogram of typical samples in negative ion mode
