# Supplementary material for: Serum organic acid metabolites can be used as potential biomarkers to identify prostatitis, benign prostatic hyperplasia, and prostate cancer
Source: Front Immunol. 2023 Jan 4;13:998447. doi: 10.3389/fimmu.2022.998447 (PMC9846500; doi:10.3389/fimmu.2022.998447)
Supplement: Supplementary file 3 [file DataSheet_3.docx]

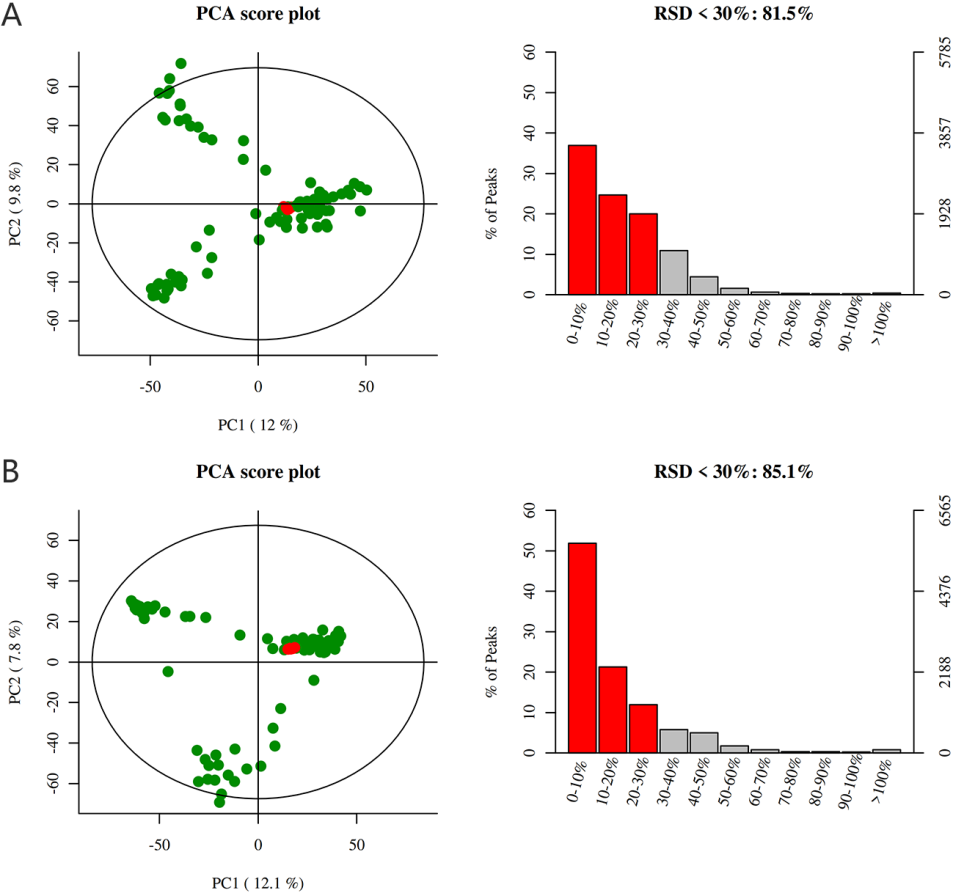


Supplemental material , Figure 3. quality assurance result chart

A: under the positive ion mode quality assurance result

B: under the negative ion mode quality assurance result chart
