# Supplementary material for: Serum organic acid metabolites can be used as potential biomarkers to identify prostatitis, benign prostatic hyperplasia, and prostate cancer
Source: Front Immunol. 2023 Jan 4;13:998447. doi: 10.3389/fimmu.2022.998447 (PMC9846500; doi:10.3389/fimmu.2022.998447)
Supplement: Supplementary file 6 [file DataSheet_6.docx]

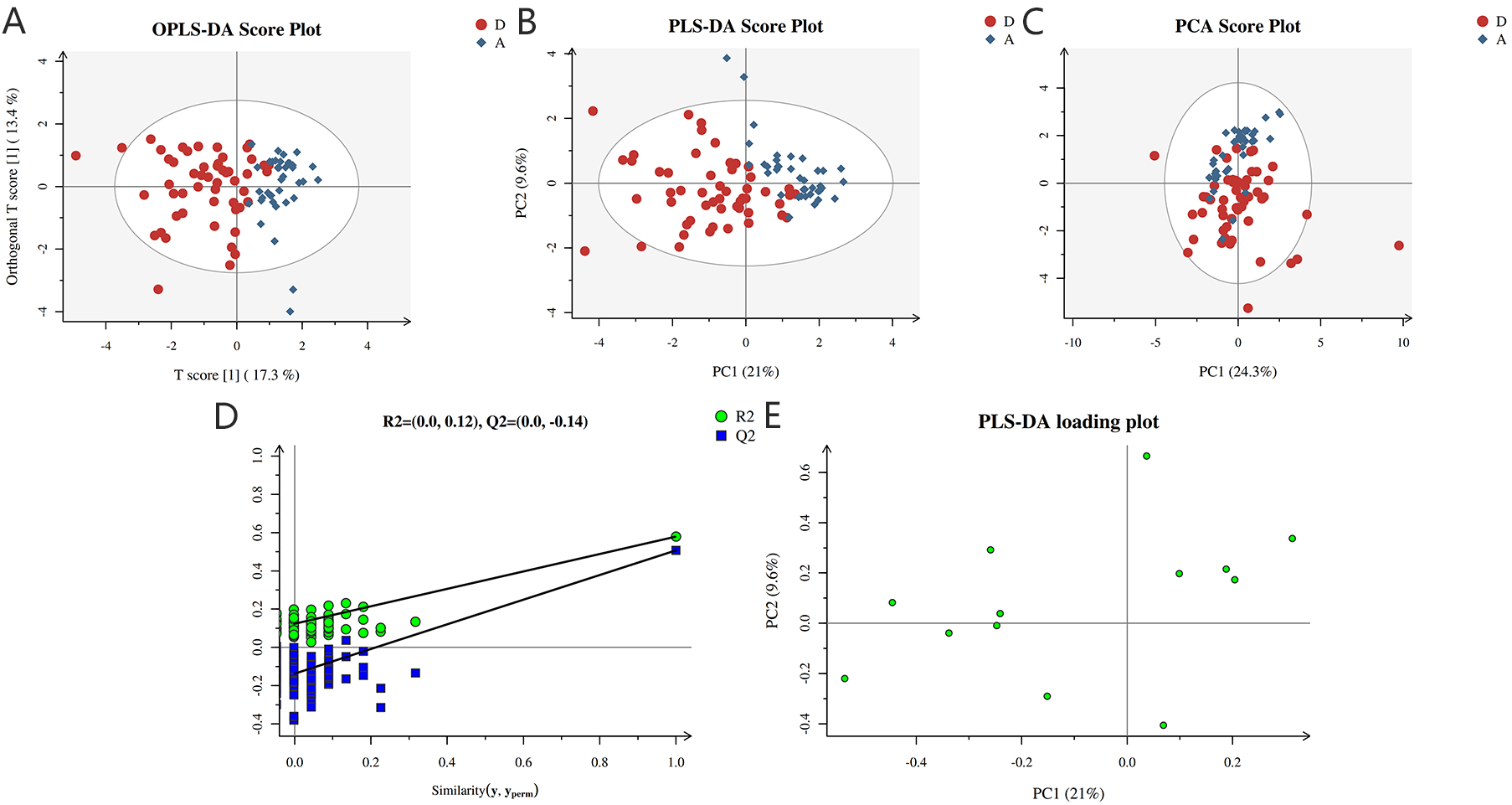


Supplemental material, Figure 6. Projections to latent structures discriminant analysis and principal component analysis

A: orthogonal Projections to Latent Structures Discriminant Analysis score

B: unsupervised analysis score

C: Principal component analysis score

D: Projections to Latent structures discriminant analysis displacement check chart

E: Projections to Latent structure discriminant analysis loading plot

Group A: control

Group D : prostate cancer
