# Supplementary material for: Serum organic acid metabolites can be used as potential biomarkers to identify prostatitis, benign prostatic hyperplasia, and prostate cancer
Source: Front Immunol. 2023 Jan 4;13:998447. doi: 10.3389/fimmu.2022.998447 (PMC9846500; doi:10.3389/fimmu.2022.998447)
Supplement: Supplementary file 7 [file DataSheet_7.docx]

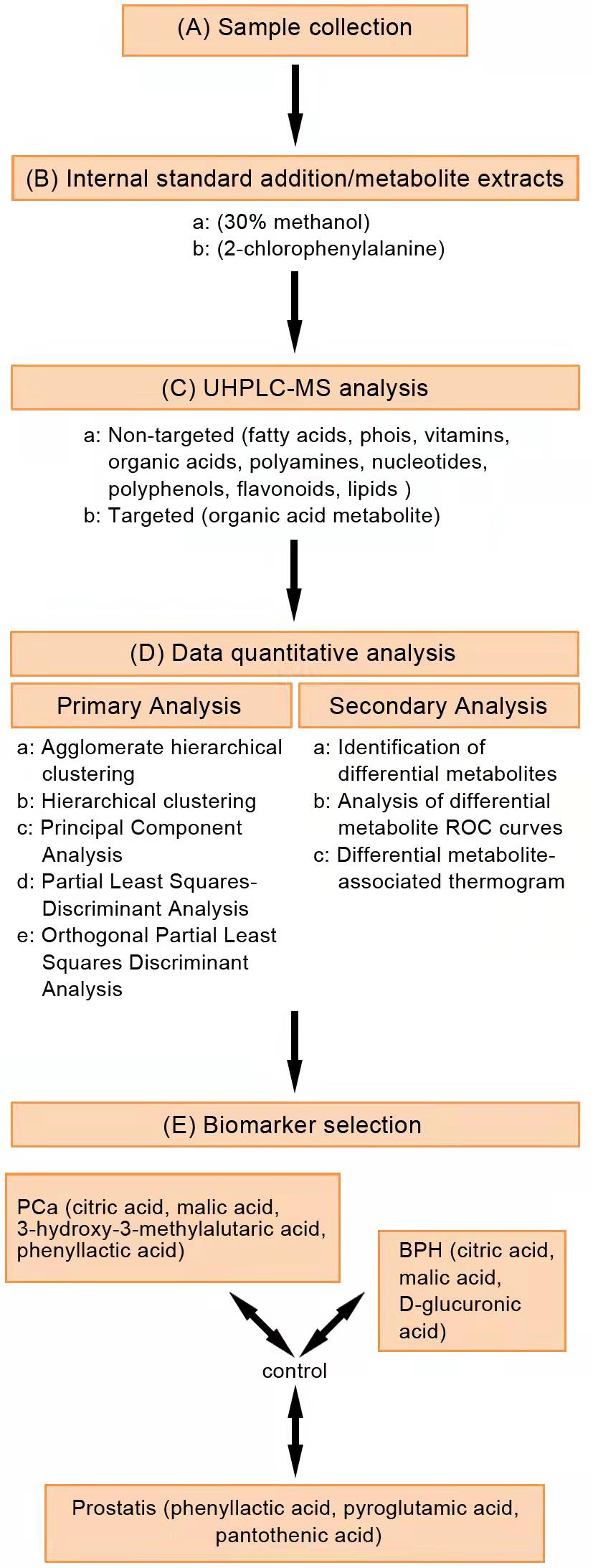


Fig. 7. Metabolic analysis workflow. (A) Sample collection. (B) Metabolite extraction/internal standard setting, using organic solvents to extract the organic matter from the sample. (C) Fatty acids, organic compounds, vitamins, and nucleic acids in serum were analyzed by non-targeted UHPLC-MS for identification. Targeted UHPLC-MS was used to identify organic acid metabolites in serum. (D) Quantitative analysis of data (including primary and secondary analysis). (E) Selection of the best candidate biomarkers for the differential diagnosis of prostatitis, BPH, and prostate cancer.
