# Supplementary material for: Serum organic acid metabolites can be used as potential biomarkers to identify prostatitis, benign prostatic hyperplasia, and prostate cancer
Source: Front Immunol. 2023 Jan 4;13:998447. doi: 10.3389/fimmu.2022.998447 (PMC9846500; doi:10.3389/fimmu.2022.998447)
Supplement: Supplementary file 10 [file Table_3.docx]

**Table 3** AUC was a metabolite of 1 in patients with prostate cancer compared with normal subjects

| **Metabolite** | **AUC** | **Ci1** | **Ci2** | **specificity** | **sensitivity** | **threshold** |
| --- | --- | --- | --- | --- | --- | --- |
| 16-Hydroxyhexadecanoic acid | 1 | 1 | 1 | 1 | 1 | 1251447.40029615 |
| 2-Heptanone | 1 | 1 | 1 | 1 | 1 | 4010038.70566441 |
| 3-Indoleacrylate | 1 | 0.986 | 1 | 0.95 | 1 | 69846275.3252918 |
| 4-Guanidinobutanoic acid | 1 | 1 | 1 | 1 | 1 | 16078084.8422784 |
| 9,10-Epoxyoctadecenoic acid | 1 | 0.986 | 1 | 0.95 | 1 | 35577202.4525972 |
| Aflatoxin B1 | 1 | 1 | 1 | 1 | 1 | 620184.870569903 |
| Alpha-D-Glucose | 1 | 1 | 1 | 1 | 1 | 2462081.44361909 |
| Azelaic acid | 1 | 1 | 1 | 1 | 1 | 7062372.7888301 |
| cis-4-Hydroxy-D-proline | 1 | 1 | 1 | 1 | 1 | 27504817.9783955 |
| Cyclic GMP | 1 | 1 | 1 | 1 | 1 | 1250115.1458596 |
| D-Mannose | 1 | 1 | 1 | 1 | 1 | 81558363.7373421 |
| Dehydroepiandrosterone sulfate | 1 | 1 | 1 | 1 | 1 | 313051.189060452 |
| Deltaline | 1 | 1 | 1 | 1 | 1 | 175593.887454517 |
| Dodecanedioic acid | 1 | 0.99 | 1 | 0.95 | 1 | 75910595.5998823 |
| Folic acid | 1 | 1 | 1 | 1 | 1 | 819449.772137513 |
| Gabapentin | 1 | 1 | 1 | 1 | 1 | 36139197.2236574 |
| Guanine | 1 | 1 | 1 | 1 | 1 | 2420241.43286011 |
| Hexadecanedioate | 1 | 1 | 1 | 1 | 1 | 153737.125419922 |
| Hydrocinnamic acid | 1 | 1 | 1 | 1 | 1 | 31934.8120497794 |
| Hydroquinone | 1 | 1 | 1 | 1 | 1 | 10876464.5556357 |
| L-Arabinose | 1 | 1 | 1 | 1 | 1 | 1504957.28386063 |
| L-Theanine | 1 | 1 | 1 | 1 | 1 | 99778.5959475214 |
| L-Tyrosine | 1 | 1 | 1 | 1 | 1 | 70262006.334519 |
| Maleic acid | 1 | 0.99 | 1 | 0.95 | 1 | 47788718.2114512 |
| N-Acetylornithine | 1 | 1 | 1 | 1 | 1 | 4671926.47573985 |
| Phenyl acetate | 1 | 1 | 1 | 1 | 1 | 78141356.1161812 |
| Phenylethylamine | 1 | 1 | 1 | 1 | 1 | 1098926.8816066 |
| Putrescine | 1 | 0.978 | 1 | 0.95 | 1 | 22336241.8662642 |
| Pyroglutamic acid | 1 | 1 | 1 | 1 | 1 | 304868665.545954 |
| Quinolinic acid | 1 | 0.811 | 1 | 0.95 | 1 | 2430123.66585505 |
| S-Carboxymethyl-L-cysteine | 1 | 1 | 1 | 1 | 1 | 2336053.9552032 |
| Succinic acid | 1 | 0.986 | 1 | 0.95 | 1 | 28658087.9716931 |
| Tamoxifen | 1 | 0.986 | 1 | 0.95 | 1 | 37097343.1399719 |
| trans-Cinnamate | 1 | 1 | 1 | 1 | 1 | 267058.014718624 |
