# Supplementary material for: Serum organic acid metabolites can be used as potential biomarkers to identify prostatitis, benign prostatic hyperplasia, and prostate cancer
Source: Front Immunol. 2023 Jan 4;13:998447. doi: 10.3389/fimmu.2022.998447 (PMC9846500; doi:10.3389/fimmu.2022.998447)
Supplement: Supplementary file 9 [file Table_1.docx]

Table 1 AUC was a metabolite of 1 in patients with prostatitis compared normal subjects

Metabolite	AUC	Ci1	Ci2	specificity	sensitivity	threshold
1,3,7-Trimethyluric acid	1	0.985	1	0.95	1	850001.2998953
1-Naphthylamine	1	1	1	1	1	1221583.344798
16-Hydroxy hexadecanoic acid	1	1	1	1	1	654264.2471763
9,10-Epoxyoctadecenoic acid	1	1	1	1	1	31327313.92959
Alpha-D-Glucose	1	1	1	1	1	1973582.65652
Citrulline	1	0.976	1	0.95	1	9370884.72412
D-Mannose	1	1	1	1	1	53517163.8618
Dehydroepiandrosterone sulfate	1	1	1	1	1	20235053.05331
Ethylmethylacetic acid	1	1	1	1	1	2829568.064804
Folic acid	1	1	1	1	1	169263.9418348
Hexadecanedioate	1	1	1	1	1	109116.0546673
