# Supplementary material for: Serum organic acid metabolites can be used as potential biomarkers to identify prostatitis, benign prostatic hyperplasia, and prostate cancer
Source: Front Immunol. 2023 Jan 4;13:998447. doi: 10.3389/fimmu.2022.998447 (PMC9846500; doi:10.3389/fimmu.2022.998447)
Supplement: Supplementary file 10 [file Table_2.docx]

Table2 AUC was a metabolite of 1 in patients with benign prostatic hyperp[illegible] compared with normal subjects

Metabolite	AUC	Ci1	Ci2	specificity	sensitivity	threshold
1-Aminocyclopropanecarboxylic acid Adenine	1	1	1	1	1	5096931.594
Estrone glucuronide	1	1	1	1	1	393.06628863[illegible]37
Glyceric acid	1	0.99	1	0.95	1	24827454.972[illegible]3
Hexadecanedioate	1	1	1	1	1	14365.440827[illegible]49
Hydrocinnamic acid	1	1	1	1	1	21457.353797[illegible]17
Phenylethylamine	1	1	1	1	1	7407381.4896[illegible]4
Sphingosine	1	0.981	1	0.95	1	19850437.422[illegible]9
